# Infants’ Dermal Exposure to Phthalates from Disposable Baby Diapers and Its Association with DNA Oxidative Damage

**DOI:** 10.3390/toxics13030218

**Published:** 2025-03-17

**Authors:** Xi Lai, Jiang Zhu, Yangyang Liu, Shengtao Ma, Meiqing Lin, Yan Hu, Jingjing Liang, Yanyan Song, Wenyan Li, Tianxin Zhao

**Affiliations:** 1 Department of Child Health Care, Guangzhou Women and Children’s Medical Center, Guangzhou Medical University, Guangdong Provincial Clinical Research Center for Child Health, Guangzhou 510623, China; 2 Guangdong-Hong Kong-Macao Joint Laboratory for Contaminants Exposure and Health, Guangdong Key Laboratory of Environmental Catalysis and Health Risk Control, Institute of Environmental Health and Pollution Control, School of Environmental Science and Engineering, Guangdong University of Technology, Guangzhou 510006, China; 3 Department of Respiratory, Guangzhou Women and Children’s Medical Center, Guangzhou Medical University, Guangdong Provincial Clinical Research Center for Child Health, Guangzhou 510623, China; 4 Department of Urology, Guangzhou Women and Children’s Medical Center, Guangzhou Medical University, Guangdong Provincial Clinical Research Center for Child Health, Guangzhou 510623, China

**Keywords:** phthalates, newborn babies, dermal exposure, disposable baby diapers, 8-OHdG

## Abstract

Phthalates are widely used plasticizers that can leach from consumer products and pose potential health risks, particularly to infants whose developing systems are vulnerable to environmental toxicants. While various exposure pathways have been identified, the contribution of dermal absorption from disposable diapers remains inadequately characterized. This study recruited 66 infants from Guangzhou, a representative city in southern China. Paired disposable diaper and urine samples were collected from each participant. Six phthalates in the diapers and nine metabolites in the urine were quantitatively analyzed. The predominant phthalate detected in the diapers was bis-2-ethylhexyl phthalate (DEHP, with a median concentration of 1670 ng/g, range: 678–5200 ng/g), followed by di-n-butyl phthalate (DnBP, 948 ng/g, range: 189–5980 ng/g), di-iso-butyl phthalate (DiBP, 333 ng/g, range: 16.1–4910 ng/g), and diethyl phthalate (DEP, 252 ng/g, range: 116–3350 ng/g). In urine, metabolites of DEHP (mEHP, mEHHP, and mEOHP) were the most abundant (87.1 ng/mL), followed by mnBP (metabolites of DnBP, 44.6 ng/mL), mEP (metabolites of DEP, 33.7 ng/mL), and miBP (metabolites of DiBP, 13.9 ng/mL). A positive correlation was observed between DnBP levels in diapers and mnBP levels in urine (r = 0.259, *p* = 0.035). Additionally, several urinary metabolites (miBP, mnBP, and mEP) were positively associated with a biomarker of DNA oxidative damage, 8-hydroxydeoxyguanosine (r = 0.265–0.316, *p* < 0.01). The estimated daily uptake of DEP, DiBP, DnBP, and DEHP through dermal absorption from diapers accounted for 44.9%, 19.5%, 15.1%, and 7.76% of total exposure to these phthalates, respectively. These findings suggest that dermal absorption from diapers is a significant exposure pathway for infants. Given that both the amount of exposure and the contribution of dermal uptake are higher in younger infants, further attention is warranted to understand the potential effects of transdermal phthalate exposure on infant growth and development.

## 1. Introduction

Phthalates are widely used as plasticizers in consumer products and are known to leach into biological systems through various exposure pathways, including dermal contact [1,2]. For infants, dermal contact represents a significant exposure route due to the frequent use of personal care products (PCPs) [3,4,5]. Phthalates have been detected in baby care products, such as infant lotions, powders, and diapers, at concentrations reaching the milligram-per-gram scale [3,6,7,8]. Notably, Park et al. reported that phthalate levels in diapers collected from markets in various countries were significantly higher than those found in paper cups, packaging films, and other commercial plastic products [9]. Ishii et al. further demonstrated that phthalates such as di-n-butyl phthalate (DnBP) and bis-2-ethylhexyl phthalate (DEHP) can migrate from diaper materials into artificial sweat, highlighting the potential for transdermal absorption [10]. Given the direct and prolonged skin contact with diapers, toxic substances released from these products can penetrate the skin and potentially harm infant health [11,12]. Emerging evidence has linked phthalate exposure to adverse health outcomes in infants, including impaired motor and mental development, increased internalizing symptoms, and higher risks of attention-deficit/hyperactivity disorder following prenatal exposure to certain DEHP metabolites [13,14].

The mechanism of transdermal phthalate exposure involves the penetration of these compounds through the stratum corneum, facilitated by their lipophilic nature and low molecular weight (LMW) [15,16]. Molecular weight significantly influences the dermal absorption rates of phthalates, with LMW phthalates such as DEP and DnBP penetrating the skin more efficiently than high-molecular-weight compounds like DEHP. Studies have reported skin permeation rates of ~2 µg/cm^2^/h for DEP, ~0.1 µg/cm^2^/h for DnBP, and only ~0.0002 µg/cm^2^/h for DEHP [17]. The permeation process begins with penetration through the lipid-rich stratum corneum, followed by diffusion through the viable epidermis, where esterases metabolize phthalates to monoesters before entering systemic circulation [18,19] Additionally, the physical state of phthalates affects absorption, with emulsified forms showing enhanced permeation compared to neat substances—a particularly relevant consideration for infant exposure through diapers where urine may facilitate this process [20].

Once absorbed into systemic circulation, phthalates and their metabolites have been shown to induce the generation of reactive oxygen species (ROS), which can overwhelm cellular antioxidant defenses and lead to oxidative damage to DNA, proteins, and lipids [21,22]. Specifically, ROS can attack guanine bases in DNA, resulting in the formation of 8-hydroxydeoxyguanosine (8-OHdG), a well-established biomarker of oxidative DNA damage [23]. However, studies specifically examining the contribution of dermal exposure from diapers to the total body burden of phthalates and its association with DNA damage remain limited.

Therefore, in our study, diapers and matched urine samples were collected from individual babies. The phthalates in diapers as well as their corresponding metabolites in urine samples were identified and quantified. The contribution of dermal exposure via skin contact with diapers to the total infant exposure to phthalates was investigated, and the risk of infant phthalate exposure in the early stage was evaluated. The urinary concentration of 8-OHdG was also quantified to partially reflect the potential impact of phthalate in diapers on newborn health.

## 2. Materials and Methods

### 2.1. Study Population and Sample Collection

Subjects were enrolled between May 2021 and October 2021 at a child healthcare department in Guangzhou serving ~30,000 patients annually. The demographic characteristics of the subjects are given in Table 1. Diapers of the same brand and style used by babies in the last week were obtained from the baby’s parents or guardian. Paired spot urine samples were also obtained from infants using disposable baby urine bags. The urine specimen was transferred to 50 mL high-density polyethylene collection bottles and shipped to the laboratory within 24 h and then immediately stored at −80 °C until final analysis. The diaper samples were wrapped with aluminum foil paper and stored at 4 °C in a refrigerator. Written informed consent and a questionnaire to obtain information on PCPs usage for each child were obtained from the parents/guardians. This research was examined and approved by the Ethics Committee of Guangzhou Women and Children’s Medical Center (No. 2022138A01).

### 2.2. Chemical Analysis

For the analysis of phthalates in baby diapers, a small part (~1 cm^2^) of the inner layer, which is in direct contact with the skin of the baby’s body, was cut using stainless steel scissors and then weighed. Subsequently, 100 mg of the diaper was treated via ultrasonic extraction with 5 mL of ethyl acetate for 30 min; this extraction was repeated twice, and the combined extracts were condensed. Then, 1 mL was taken for the final instrumental analysis after the addition 20 ng of benzyl benzoate as the internal standard. Gas chromatography (GC) coupled with tandem mass spectrometry operated in the electron impact ionization mode (GCMS-TQ8040, Shimadzu, Japan) was applied to analyze the six target phthalates in the sample extracts. Urinary metabolites of phthalates (mPAEs) were analyzed via a method described earlier [24]. The determination of 8-OHdG in the urine was carried out according to the method reported previously [25]. Full details for the chemical analysis are given in Text S1 in the Supplementary Materials.

### 2.3. Statistical Analysis

The statistical analysis was only performed for targets with detection frequencies (DFs) no less than 50% by using SPSS (Version 13.0, Chicago, IL, USA). The targets with concentrations below the instrument detection limit (IDL) were assigned a value of zero, while targets with concentrations higher than the IDL but lower than the method detection limit (MDL) were displaced by MDL/2. To determine whether the concentration values of these targets obeyed a normal distribution, the Kolmogorov–Smirnov test was used. The correlations between the external and internal exposure were evaluated using Spearman’s correlation analysis when these data failed to obey a normal distribution. A *p*-value < 0.05 was considered for statistical significance in all analyses.

## 3. Results and Discussion

### 3.1. Phthalates in Diapers

Among the six target phthalates investigated, four phthalates, namely DEP, DnBP, DiBP, and DEHP, were detected in all of the diaper samples (Table 2), while the other phthalate isomers were non-detectable. The total concentrations of phthalates (∑PAEs) in the diapers ranged from 1870 to 12,500 ng/g, with a median value of 3420 ng/g. DEHP was the predominant isomer (median level of 1670 ng/g), accounting for 48.8% of ∑PAEs, followed by DnBP (948 ng/g), DiBP (333 ng/g), and DEP (252 ng/g). The phthalate concentrations in our study were higher than those found in diapers from Japan [10] and six other countries (the United States, Finland, France, Greece, Korea, and Japan) [9]. Ishii et al. reported that two of the seven phthalates (i.e., DEHP and DnBP) were detectable in the top sheets of paper diapers, and the DEHP concentration was higher than the DnBP concentration [10], which is consistent with our findings. Park et al. reported that DnBP (ranging from 13.4 to 1610 ng/g) was the predominant phthalate in four different brands of diapers investigated, with a higher concentration than DEHP (12.6–62.8 ng/g) [9], which was contrary to our results. Although only two studies have investigated the occurrence of phthalates in diapers, our study and previous studies indicate that diapers are highly contaminated with phthalates. In addition, the concentrations of phthalates found in diapers from our study were slightly higher or in the same ranges as those found in sanitary napkins worldwide [6,8,26], as well as panty liners and pads from the United States [7] (Table 2). The composition of phthalates in diapers was consistent with the major commercial products consumed in China [1], suggesting that the phthalates might have originated from raw materials used for diaper product manufacturing.

### 3.2. Phthalate Metabolites in Urine

The monoalkyl metabolites of DEP, DnBP, DiBP, and DEHP were found in most of the urine samples from infants, with a DF of 100% for miBP, mBP, mEHP, and mEOHP and DFs of 89% and 96% for mEP and mEHHP, respectively (Table 3). However, mBzP and mMP were non-detectable in all of the urine samples. The high DFs of the urinary metabolites of phthalates indicated that the infants were widely exposed to some phthalates.

The urinary concentration of total mPAEs ranged from 14.8 to 1860 ng/mL, with a median of 238 ng/mL. The sum concentrations of the metabolites of DEHP (namely mEHP, mEHHP, and mEOHP) exhibited the highest level, with a median of 87.1 ng/mL, followed by mnBP (44.6 ng/mL), mEP (33.7 ng/mL), and miBP (13.9 ng/mL). The metabolites of DEHP accounted for 41.5% of the total metabolites of phthalates (∑mPAEs), while mnBP, mEP, and miBP accounted for 29.8%, 20.2%, and 8.5% of ∑mPAEs, respectively. The composition profiles of the urinary phthalate metabolites also agreed well with the corresponding phthalates found in matched diapers.

The concentrations of mPAEs were in the same range as those from children from southern China reported by Ma et al. (11.5–1750 ng/mL) [24] and Yu et al. (9.48–1609 ng/mL) [27], with a similar composition profile. The urinary concentrations of mPAEs in infants in our study were also higher than those for infants aged 6–14 months from Korea [28], as well as children aged 3–6 years from Japan [29]. In addition, relatively higher mEP levels were observed in the urine of infants in our study, attributable to the extensive DEP addition in baby care products, as DEP has been the most frequently detected phthalate in baby oils, shampoos, lotions, and diaper creams [15]. Predominant mEP has also been found in the urine of children from Saudi Arabia [30], Brazil [22], Spain [31], and the U.S. [32], which may be relevant to the different phthalate-containing products consumed. In addition, mEHP, a primary hydrolysis product of DEHP, was found as the predominant urinary metabolite in our study; while the other two secondary oxidative monoesters (mEHHP and mEOHP) of DEHP were found as major contributors in southern China [24,27] and other countries (Table S5) [22,28,29,31]. For breastfed infants less than one year old, the ingestion of phthalates from breast milk also influences the composition of the metabolites of DEHP in the body, as studies have indicated that mEHP was almost exclusively detected in breast milk samples [33,34]. Further statistical analysis showed that the urinary concentration of mEHP in breastfed infants (<1 year old, *n* = 29; median = 48.1 ng/mL) was higher than in non-breastfed infants (>1 year old, *n* = 37; median = 27.7 ng/mL, Mann–Whitney *U* test, *p <* 0.05), which was in line with the above speculation. Because a developing child (fetus to prepuberty) is particularly susceptible to environmental influence, more concern should be aroused to the potential adverse impact of the high level of phthalate exposure in infants.

### 3.3. Correlations Between Phthalates in Diapers and Their Metabolites in Urine

Spearman’s correlation tests indicated that DiBP, DnBP, and DEHP were positively correlated with each other (*r* = 0.324–0.655, *p* < 0.01), while no correlation was found between DEP and other phthalates. The differences might be related to the use of different phthalate-containing commercial products, as DEP is generally used as an essence solvent in PCPs [1,2], while DiBP, DnBP, and DEHP might be incorporated into polypropylene non-woven fabric, which is used for the manufacture of the top sheet of diapers [10].

For the metabolites detected in urine, the three DEHP metabolites were positively correlated (*r* = 0.408–0.983, *p* < 0.01). In particular, an almost linear correlation was observed for two oxidative metabolites of mEHP (mEOHP and mEHHP, *r* = 0.983, *p* < 0.01), possibly because these metabolites were derived from the secondary oxidative product of the same precursor, mEHP, in agreement with reports in the literature [1,24].

For paired diapers and urine samples, the concentrations of DnBP in diapers and the urinary levels of mnBP were weakly correlated (*r* = 0.259, *p* = 0.035, Spearman’s correlation test, Figure S1). A weak correlation was also found between the concentrations of DnBP in diapers and levels of miBP in urine samples (*r* = 0.297, *p* = 0.015), attributable to the significant correlation between miBP and mnBP in urine samples. No significant correlation existed between the concentration of DEHP in diapers and the levels of its metabolites in urine, although DEHP and its metabolites were identified as the predominant isomers. The relatively large molecular weight of DEHP might further hinder its penetration of the skin and stratum corneum. To date, no reports have determined the association of internal metabolites with dermal exposure using paired urine and diaper samples. Our results suggest that dermal absorption resulting from diaper usage represents a potential, though often minor, pathway of infant exposure to phthalates, with DnBP showing the most evident association between external and internal exposure.

### 3.4. Associations Between Urinary Phthalate Metabolite Levels and 8-OHdG Concentrations

8-OHdG was detected in all of the urine samples with a mean concentration of 1.58 ± 0.84 ng/mL. No significant gender differences were found. Spearman’s correlation analysis showed that 8-OHdG concentrations were significantly correlated with mEP, miBP, mnBP, and mPAEs in urine, with correlation coefficients of 0.265 (*p* = 0.031), 0.316 (*p* = 0.010), 0.297 (*p* = 0.016), and 0.318 (*p* = 0.009) (Table 3), respectively. In addition, the concentration of urinary 8-OHdG was positively correlated with DnBP concentrations in diapers (r = 0.326, *p* = 0.038), suggesting that there may be an association between infant dermal exposure to phthalates in diapers and oxidative damage to DNA.

No studies have directly examined the impact of diaper-derived phthalate exposure on oxidative stress biomarkers, but similar effects have been observed with other endocrine-disrupting chemicals. For example, Lv et al. reported that higher dermal exposure to bisphenol A in cashiers was linked to elevated urinary 8-OHdG levels [35]. Oxidative stress biomarkers like 8-OHdG may also be influenced by phthalate alternatives, such as non-phthalate plasticizers like diisononyl cyclohexane-1,2-dicarboxylate [36], and other toxic compounds found in diapers, including formaldehyde, benzene, toluene, dioxins, and polycyclic aromatic hydrocarbons [12]. Beyond diapers, infants are exposed to complex chemical mixtures from air, food, and personal care products, which may collectively contribute to oxidative stress.

Our findings suggest that infant dermal exposure to phthalates through diapers may lead to oxidative DNA damage, warranting further large-scale investigations. Persistent oxidative stress during early development could impair cellular function and increase the risk of chronic diseases later in life, such as neurodevelopmental disorders and metabolic conditions [37]. Given infants’ vulnerability due to immature detoxification systems and rapid growth, understanding these mechanisms is critical for assessing health risks associated with phthalate exposure.

### 3.5. Assessment of Daily Intake (DI) and Human Health Risks

The estimated daily intake of phthalates is given in detail in the Supplementary Materials (Text S2). As shown in Table 4, the DI of phthalates that newborn babies were exposed to by wearing diapers ranged from 21.2 to 1220 ng/kg-bw/day, with a median of 159 ng/kg-bw/day. The median DI decreased in the following order: DnBP (61.7 ng/kg-bw/day) > DEHP (46.3 ng/kg-bw/day) > DiBP (23.1 ng/kg-bw/day) > DEP (19.6 ng/kg-bw/day) (Table 4). Infants less than 6 months old were exposed to the highest levels of phthalates through dermal contact with diapers, with medians of 61.3 ng/kg-bw/day for DEP, 38.3 ng/kg-bw/day for DiBP, 130 ng/kg-bw/day for DnBP, and 117 ng/kg-bw/day for DEHP. For infants over 2 years old, the values were 7.2, 21.3, 29, and 22.2 ng/kg-bw/day, respectively, possibly because younger babies have their diapers changed more frequently than older babies. The DI of phthalate found in diapers in this study was higher than those reported in Japan (4.9 ng/kg-bw/day) [10].

According to the levels of urinary phthalate metabolites, the total daily intake (TDI) of phthalates was calculated, with medians of 1890 ng/kg-bw/day for DEP, 1090 ng/kg-bw/day for DiBP, 3510 ng/kg-bw/day for DnBP, and 47,100 ng/kg-bw/day for DEHP. The DI-to-TDI ratio was applied to represent the proportion of the daily dermal absorption from diapers in terms of the total daily intake of phthalates, with median values of 0.91% for DEP, 1.88% for DiBP, 2.22% for DnBP, and 0.11% for DEHP. Generally, phthalate exposure via dermal contact with diapers contributed little to the total body burden, which partly explains why the concentrations of these phthalate metabolites in urine show a weak correlation with the phthalates in matched diapers. However, the estimated intakes of DEP, DiBP, DnBP, and DEHP from diapers at the 95th percentile approximately contributed 44.9%, 19.5%, 15.1%, and 7.76% to the total exposure, respectively, suggesting that diapers are one of the exposure sources of these chemicals.

The median hazard quotients (HQs) for each phthalate were 0.063 for DEP, 0.109 for DiBP, 0.351 for DnBP, and 0.094 for DEHP. The estimated median HI for the infants was 0.86, and 37.9% of the infants were considered at risk (HI > 1), suggesting that the risk of neonatal exposure to phthalates through diapers was nonnegligible.

## 4. Conclusions and Limitations

This study revealed widespread phthalate contamination in disposable diapers and suggests that dermal absorption represents a potential exposure pathway for infants, particularly younger ones due to more frequent diaper changes. We observed a positive correlation between DnBP levels in diapers and mnBP levels in urine, as well as associations between urinary phthalate metabolites and the oxidative stress biomarker 8-OHdG, warranting further investigation into the potential effects of phthalate exposure on infant health.

Several limitations should be considered when interpreting our findings. First, we did not normalize urinary metabolite concentrations with creatinine values, which may lead to over- or underestimation of actual exposure levels due to variations in urine dilution. Second, while we focused on diaper-derived exposure, infants are simultaneously exposed to phthalates through multiple pathways including diet, indoor air, dust, and personal care products, making it challenging to isolate the specific contribution from diapers. Third, our cross-sectional design cannot establish causality between phthalate exposure and oxidative DNA damage. Finally, our sample size was relatively modest and drawn from a single urban location, potentially limiting the generalizability of our findings to other populations with different diaper usage patterns and exposure profiles.

## Figures and Tables

**Table 1:** Characteristics of subjects.

Characteristic	Frequency (%)	Mean ± SD ^a^	Median (Range)
Sex, n (%)			
Male	53 (80.3)		
Female	13 (19.7)		
Age, years		1.28 ± 0.99	1.16 (0.1–4.0)
≤0.5	21 (31.8)	0.24 ± 0.11	
0.5–1.0	10 (15.2)	0.80 ± 0.14	
≥1.0	35 (53.0)	2.1 ± 0.71	
Frequency of daily diaper changing		5.33 ± 3.69	3 (1–13)
Age ≤ 0.5 year		10.3 ± 1.3	
Age 0.5–1.0 year		5.2 ± 1.4	
Age ≥ 1.0 year		2.4 ± 0.7	
Body mass index, kg/m^2^		16.46 ± 1.71	16.61 (13.15–20.76)

^a^ SD: standard deviation.

**Table 2:** Concentrations of phthalates in diapers and comparison with other personal care products.

Items	Phthalate Contents in the Inner Layer of Diaper (ng/g)					Reference
	DEP	DiBP	DnBP	DEHP	∑PAEs	
Diaper from southern China (n = 66, 2021)						
Median (range)	252 (116–3350)	333 (16.1–4910)	948 (189–5980)	1670 (678–5200)	3420 (1870–12,500)	This study
Mean	309 ± 386	680 ± 908	1270 ± 904	1830 ± 934	4090 ± 2150	
Detection frequency	100%	100%	100%	100%	100%	
Diapers from Japan (n = 10)						
Median (range)	n.a.	<LOD	(100–200)	(200–600)	n.a.	[10]
Diapers from six countries (n = 12)						
Median (range)	(0.8–2.9)	n.a.	(13.4–1610)	(12.6–62.8)	n.a.	[9]
Sanitary napkins from six countries (n = 48)						
Median (range)	(<LOD–134)	n.a.	(52.1–7820.4)	(5.5–197.4)	n.a.	[9]
Sanitary napkins from China (n = 64, 2017–2018)						
Median (range)	80 (<LOD–1710)	230 (<LOD–1590)	240 (<LOD–2380)	440 (<LOD–8040)	1430 (250–8760)	[8]
Panty liners from New York, the United States (n = 13, 2019)						
Median (range)	386 (45.6–1070)	299 (25.1–5500)	393 (21.3–6070)	164 (11.1–23,400)	1830 (168–34,500)	[7]
Sanitary napkins from New York, the United States (n = 18, 2019)						
Median (range)	82 (50.9–1200)	73 (25.9–5400)	83.3 (22.0–3630)	38.7 (14.9–858)	362 (205–11,200)	[7]
Sanitary napkins from six countries (n = 72)						
Median (range)	n.a.	905	711	822	1859 (464–8380)	[6]
Sanitary napkins from China (n = 40, 2016)						
Median (range)	n.a.	1270	991	1086	4974 (2705–13,779)	[26]

∑PAEs (phthalate esters), DEP (diethyl phthalate), DiBP (di-iso-butyl phthalate), DnBP (di-n-butyl phthalate), and DEHP (bis-2-ethylhexyl phthalate); <LOD means concentrations were below the limit of quantification; n.a. means not available.

**Table 3:** Urinary concentrations and correlations of phthalate metabolites (mPAEs) and 8-OHdG in infants.

Parent Compound	Metabolite	DF ^a^ (%)	Mean ± SD	Median	95th ^b^	Range	R (*p*) ^c^
DEP	mEP	89	65.5 ± 80.4	33.1	229	<MDL–386	0.265 (0.031)
DiBP	miBP	100	17.4 ± 13.8	13.9	44	0.75–71.3	0.316 (0.01)
DnBP	mnBP	100	60.2 ± 68.8	44.6	196	3.99–451	0.297 (0.016)
DEHP	mEHP	100	70.6 ± 136	27.7	278	0.14–736	-
	mEHHP	96	71.9 ± 170	13.8	240	<MDL–1220	-
	mEOHP	100	37.2 ± 62.2	10.2	180	0.34–293	-
	∑mDEHP	100	177 ± 278	87.1	-	0.78–1750	-
	∑mPAEs	100	313 ± 309	238	-	14.8–1860	0.318 (0.009)
8-OHdG	-	100	1.58 ± 0.84	1.38	-	0.016–5.066	

^a^ Detection frequency; ^b^ 95th percentile; ^c^ Spearman’s correlation between certain phthalate metabolites and 8-OHdG.

**Table 4:** Estimated daily intake (ng/kg-bw/day) of phthalates from diapers for infants.

Item	Total (n = 66)	0−0.5 Year (n = 21)	>0.5−1.0 Year (n = 10)	>1.0−2.0 Year (n = 20)	>2.0 Year (n = 15)
DEP					
Median	19.6	61.3	26.7	14.3	7.2
Range	2.1–561	41–561	18.8–70.5	7.3–25.4	2.1–10.1
95th percentile	95.4				
DiBP					
Median	23	38.2	20.6	14.6	21.3
Range	0.18–764	2.5–764	1.1–80.2	0.67–100	0.18–188
95th percentile	178				
DnBP					
Median	61.7	130	65.9	38.2	29
Range	4.5–523	34.7–523	31.8–182	13.8–104	4.5–229
95th percentile	231				
DEHP					
Median	46.3	117	74.2	28.7	22.2
Range	6.1–349	52–349	20–131	12.6–104	6.1–40.5
95th percentile	161				
∑PAEs					
Median	159	380	211	100	79.6
Range	21.2–1220	169–1220	82.1–405	34.4–334	21.2–452
95th percentile	690				

∑PAEs (phthalate esters), DEP (diethyl phthalate), DiBP (di-iso-butyl phthalate), DnBP (di-n-butyl phthalate), and DEHP (bis-2-ethylhexyl phthalate).

## Data Availability

The data that support the findings of this study are available on request from the corresponding author, W.L. and T.Z., upon reasonable request.

## Supplementary Materials

The following supporting information can be downloaded at: https://www.mdpi.com/article/10.3390/toxics13030218/s1, Figure S1: Scatter plots of Spearman’s correlation between DnBP in diapers and urinary mnBP of infants (n = 66); Table S1: GC-MS/MS operating parameters for the analysis of phthalates; Table S2: HPLC-MS/MS operating parameters for the analysis of mPAEs.title; Table S3: Recovery, linear ranges and correlation coefficients (R2) of calibration curves, instrument detection limits (IDL) and method detection limits (MDL) of the target phthalates; Table S4: Recovery, linear ranges and correlation coefficients (R2) of calibration curves, instrument detection limits (IDL) and method detection limits (MDL) of mPAEs; Table S5: Median urinary concentrations of phthalate reported from different Children; Text S1: Chemicals analysis; Text S2: Estimated daily intake. Refs. [5,6,10,22,24,25,28,29,30,31,32,38,39,40,41] are cited in Supplementary Materials.
