# Supplementary material for: Infants’ Dermal Exposure to Phthalates from Disposable Baby Diapers and Its Association with DNA Oxidative Damage
Source: Toxics. 2025 Mar 17;13(3):218. doi: 10.3390/toxics13030218 (PMC11946139; doi:10.3390/toxics13030218)
Supplement: Supplementary file 1 [file toxics-13-00218-s001.zip › toxics-3499388-supplementary.pdf]

Supporting information

Infants' Dermal Exposure to Phthalates from Disposable Baby Diapers and Its Association with DNA Oxidative Damage

Xi Lai [1,†], Jiang Zhu [1,†], Yangyang Liu [2], Shengtao Ma [2], Meiqing Lin [2], Yan Hu [1], Jingjing Liang [1], Yanyan Song [1], Wenyan Li [3,*] and Tianxin Zhao [4,*]

[1] Department of Child Health Care, Guangzhou Women and Children's Medical Center, Guangzhou Medical University, Guangdong Provincial Clinical Research Center for Child Health, Guangzhou 510623, China
[2] Guangdong-Hong Kong-Macao Joint Laboratory for Contaminants Exposure and Health, Guangdong Key Laboratory of Environmental Catalysis and Health Risk Control, Institute of Environmental Health and Pollution Control, School of Environmental Science and Engineering, Guangdong University of Technology, Guangzhou 510006, China
[3] Department of Respiratory, Guangzhou Women and Children's Medical Center, Guangzhou Medical University, Guangdong Provincial Clinical Research Center for Child Health, Guangzhou 510623, China
[4] Department of Urology, Guangzhou Women and Children's Medical Center, Guangzhou Medical University, Guangdong Provincial Clinical Research Center for Child Health, Guangzhou 510623, China
* Correspondence: liwenyan32@126.com (W.L.); txzhaos@gwcmc.org (T.Z.)
† These authors were listed as the co-first author.

Text S1 Chemicals analysis

Six phthalates, namely DMP, DEP, DnBP, DiBP, BBzP, and DEHP, were purchased from Dr. Ehrenstotfer (Augsburg, Germany). The internal standard (benzyl benzoate) used for the analysis of phthalates was purchased from AccuStandard, Inc. (New Haven, CT, USA). Nine metabolites of the corresponding phthalates (mPAEs), namely mono-methyl phthalate (mMP) of DMP, mono-ethyl phthalate (mEP) of DEP, mono-iso-butyl phthalate (miBP) of DiBP, mono-n-butyl phthalate (mnBP) of DnBP, mono-benzyl phthalate (mBzP) of BBzP, mono-(2-ethylhexyl) phthalate (mEHP), mono-(2-ethyl-5-hydroxy-hexyl) phthalate (mEHHP), and mono-(2-ethyl-5-oxo-hexyl) phthalate (mEOHP) of DEHP, and their corresponding surrogate standards, that is, $^{13}C_4$-mMP, $^{13}C_4$-mEP, miBP-d_4, $^{13}C_4$-mnBP, $^{13}C_4$-mBzP, $^{13}C_4$-mEHP, $^{13}C_4$-mEHHP, and $^{13}C_4$-mEOHP, were purchased from CIL (Andover, MA, USA). The enzyme β-glucuronidase (E.coli K12, β-glucuronidase activity ≥ 140 U/mL) were purchased from Roche Diagnostics GmbH (Mannheim, Germany).

For the instrumental analysis of phthalates, injections of 1 μL were conducted with an AOC-20s automatic sampler in the splitless mode. The inlet temperature was maintained at 280°C. The temperatures for ion source and interface were set at 230°C and 300°C, respectively. A DB-5ms capillary column (30 m × 0.25 mm × 0.25 μm, Agilent, CA, USA) was employed for the chromatographic separation of the six target phthalates. The GC oven temperature was initially held at 60°C for 1 min, ramped to 200°C at 10°C/min, then increased to 280°C at 5°C/min, finally raised to 300°C at

10°C/min and held for 10 min. The chromatographic retention times, multiple reaction monitoring (MRM) transitions, and the collision energies were the same as in our previous report [1], with full details provided in Table S1.

Urinary metabolites of phthalates were analyzed *via* a method described earlier [1]. After adding labeled internal standards, 5 μL of β-glucuronidase (*Escherichia coli* K12, Roche, Mannheim, Germany) was added to 500 μL of urine samples. The mixture was incubated at 37°C for 12 h. Then, the urine samples were protein-precipitated through the addition of equal amounts of acetonitrile. The mixture was thoroughly mixed via shaking or vertexing for 30 s, and the precipitate was removed by centrifugation (12,000 g, 10 min). An online solid-phase extraction coupled with column-switching liquid chromatography-tandem mass spectrometry (LC-MS/MS) was used for mPAEs analysis. The instrumental analysis parameters were provided in Table S2.

The determination of 8-OHdG in urine employed a method involving several key steps [2]. First, urine samples were centrifuged, and the supernatant was treated with formic acid and isotope-labeled internal standard (8-OHdG-$^{15}N_5$). Samples then underwent overnight enzymatic hydrolysis with β-glucuronidase/arylsulfatase at 37°C. The target analyte was isolated using solid-phase extraction with Cleanert PEP cartridges, which were preconditioned, loaded with sample, and eluted with 2% formic acid-acetonitrile. After evaporation and reconstitution, the extracts were analyzed by UPLC-ESI/MS/MS using an Ultimate AQ-C18 column with a mobile

phase of 0.05% formic acid in water and methanol. Mass spectrometric detection was performed in negative ESI mode, with 8-OHdG quantified through selective reaction monitoring of the precursor ion (m/z 282) to product ion (m/z 192) transition using optimized collision parameters.

For every batch of 10 samples, one procedural blank and one spiked sample were analyzed with following exactly the same procedures as the field samples. For the analysis of phthalates in diapers, the pre-extracted diaper spiked with 200 ng of phthalates was used; while diluted urine spiked with 10 ng of mPAEs was used as the blank matrix for urine analysis. The recoveries for phthalates in diapers were ranged from 75.2% to 102% (Table S3); for mPAEs in urine were ranged from 75% to 99.8%; for 8-OHdG in urine was 92.3% (Table S4). None of these target phthalates or metabolites were detected in the procedural blank samples. The method detection limits (MDLs) were based on an analyte with signal-to-noise ratio of 5 or three times the standard deviation of the average field blank, which ranged from 1.0 to 50 ng/g for phthalates, from 0.1 to 5.0 ng/mL for mPAEs, and 0.05 ng/mL for 8-OHdG (Table S4).

Text S2 Estimated daily intake

The estimated daily intake (DI) of phthalates for infants *via* paper diapers was calculated according to a formula reported by Tang et al. [3]:

$$DI \text{ (ng/kg-bw/day)} = \frac{C \times M_d \times N_d \times A_{bs}}{b_w} \quad \text{(S1)}$$

where C is the content (ng/g) of phthalates in the inner layer of the diaper; M_d is the weight (g) of the inner layer per paper diaper, which was individually weighed; N_d is the average number of disposable diapers used per day, which was acquired using questionnaires (Table S1); A_{bs} is the transdermally absorbed rate of phthalates, with values of 5%, 10% and 10% for DEHP, DnBP, and DiBP, respectively, as given by Ishii et al. [4]; An A_{bs} of 15% was applied for DEP according to Tang et al. [3]; b_w represents the body weight (kg) of babies.

The total daily intake (TDI) was calculated according to the following formula [5]:

$$TDI \text{ (ng/kg-bw/day)} = \frac{C_{\mathrm{m}} \times V \times \mathrm{MW_p}}{f \times \mathrm{MW_m}} \quad \text{(S2)}$$

where C_{m} is the concentration of mPAEs in urine samples (ng/mL); V represents the average daily urinary excretion volume (mL/day), which was calculated based on 44 mL/kg body weight per day for infants according to Völkel et al. [5]; $\mathrm{MW_p}$ represents the molecular weight of the target phthalate (g/mol); $\mathrm{MW_m}$ represents the molecular weight of the corresponding mPAE (g/mol); and f represents the molar excretion fraction of mPAE in urine according to the ingestion dosage of the parent phthalate. The f values were supposed to be 0.69 for mEP, 0.70 for mnBP, 0.70 for miBP, 0.059 for mEHP, 0.23 for mEHHP, and 0.15 for mEOHP [6]. Moreover, the TDI of DEHP was estimated according to the total concentrations of DEHP calculated from mEHP, mEHHP, and mEOHP. The DI-to-TDI ratio was used to represent the contribution of the transdermal absorption from diapers to the total phthalate uptake.

According to formula (3), the hazards index (HI) for each infant was obtained by calculating the sum of the ratio of the TDI to the acceptable level of daily intake for each phthalate isomers (i.e., hazard quotients, HQ). The acceptable levels of daily intake phthalates were obtained from the European Food Safety Authority, as well as the reference dose (RfD) for anti-androgenicity as summarized by Ma et al. [1], with values of 30, 10, 10 and 500 μg/kg-bw/day for DEP, DiBP, DnBP, and DEHP, respectively.

$$HI = HQ_{DEP} + HQ_{DiBP} + HQ_{DnBP} + HQ_{DEHP} = \frac{TDI_{DEP}}{RfD_{DEP}} + \frac{TDI_{DiBP}}{RfD_{DiBP}} + \frac{TDI_{DnBP}}{RfD_{DnBP}} + \frac{TDI_{DEHP}}{RfD_{DEHP}} \quad (S3)$$

Table S1. GC-MS/MS operating parameters for the analysis of phthalates.

Chemical	Retention time (min)	Quantifier (Collision energy)	Qualifier (Collision energy)
DMP	12.105	163.0 > 77.1 (24 eV)	163.0 > 133.1 (9 eV)
DEP	13.840	149.0 > 65.1 (27 eV)	149.0 > 93.1 (18 eV)
Benzyl benzoate (IS)	16.060	105.0 > 77.1 (15 eV)	105.0 > 51.1 (24 eV)
DiBP	17.085	149.0 > 121.1 (15 eV)	223.0 > 149.1 (18 eV)
DnBP	18.335	149.0 > 121.1 (15 eV)	149.0 > 93.1 (18 eV)
BBzP	23.980	149.0 > 65.1 (27 eV)	149.0 > 93.1 (18 eV)
DEHP	26.715	167.0 > 65.1 (33 eV)	167.0 > 149.0 (9 eV)

Table S2. HPLC-MS/MS operating parameters for the analysis of mPAEs.

Chemical	Retention time (min)	MRM transitions (Parent > Product)	Fragmentor (V)	Collision energy (eV)
mMP	6.13	179.0 > 107.2	75	15
		179.0 > 77.1	63	13
$^{13}C_4$-mMP	6.11	183.0 > 109.0	53	13
mEP	6.86	192.9 > 121.0	89	15
		192.9 > 146.9	60	14
$^{13}C_4$-mEP	6.83	196.9 > 124.0	51	25
miBP	10.55	220.9 > 134.2	51	18
		220.9 > 71.0	56	18
d_4-miBP	10.42	225.0 > 138.1	64	17
mnBP	10.83	221.0 > 70.8	62	23
		221.0 > 120.9	62	20
$^{13}C_4$-mnBP	10.80	225.2 > 124.0	71	19
mBzP	11.55	255.2 > 107.1	84	32
		255.2 > 77.3	67	24
$^{13}C_4$-mBzP	11.52	259.9 > 106.8	62	32
mEHP	19.74	277.2 > 133.8	67	22
		277.2 > 127.1	59	21
$^{13}C_4$-mEHP	19.72	281.4 > 137.1	64	21
mEHHP	9.32	293.1 > 120.9	61	28
		293.1 > 145.1	56	21
$^{13}C_4$-mEHHP	9.27	297.3 > 123.9	55	27
mEOHP	10.09	291.0 > 143.1	62	19
		291.0 > 120.8	62	19
$^{13}C_4$-mEOHP	10.06	295.2 > 143.2	62	23

Table S3. Recovery, linear ranges and correlation coefficients (R^2) of standard solutions, instrument detection limits (IDL) and method detection limits (MDL) of the target phthalates.

Compound	Linear ranges (μg mL^{-1})	R^2	IDL (pg)	MDL (ng/g)	Recovery (Mean ± SD, %)
DMP	0.05−20	0.9995	0.50	5.0	89.4±5.3
DEP	0.05−20	0.9996	0.40	4.0	86.1±4.6
DiBP	0.05−20	0.9998	0.10	1.0	91.9±4.7
DnBP	0.05−20	0.9996	0.10	1.0	82.3±5.6
BBzP	0.05−20	0.9997	5.0	50	75.2±6.1
DEHP	0.05−20	0.9999	0.10	1.0	102±9.6

Table S4. Recovery, linear ranges and correlation coefficients (R^2) of calibration curves, instrument detection limits (IDL) and method detection limits (MDL) of phthalate metabolites (mPAEs) and 8-OHdG.

Compound	Linear range (ng mL^{-1})	R^2	IDL (ng mL^{-1})	MDL (ng mL^{-1} urine)	Recovery (Mean ± SD, %)
mMP	0.5−100	0.9998	0.50	2.5	99.8±9.8
mEP	0.5−100	0.9998	0.35	1.2	89.6±5.5
miBP	0.5−100	0.9996	0.35	1.2	80.1±6.8
mnBP	0.5−100	0.9998	0.35	1.2	82.6±4.2
mBzP	0.5−100	0.9994	0.30	1.0	85.2±6.9
mEHP	0.5−100	0.9989	0.03	0.12	76.4±7.2
mEHHP	0.5−100	0.9999	0.02	0.08	75.0±6.8
mEOHP	0.5−100	0.9999	0.02	0.08	75.2±3.2
8-OHdG	0.1−50	0.9993	0.02	0.05	92.3±4.5

Table S5. Median urinary concentrations of phthalate reported from different Children.

Country	Studied population	Sampling time	Concentrations (μg L^{-1})						Reference
			mEP	miBP	mnBP	mEHP	mEHHP	mEOHP	
Southern China	Infants (n=66; 0.1–4 years)	2021	33.7	13.9	44.6	27.7	13.8	10.2	This study
Southern China	Children (n=82; 4–10 years)	2019	1.91	17.9	129	19.2	22.8	13.4	[1]
Southern China	Children (n=166; 8–11 years)	2015	0.22	25.3	63.6	11.1	38.9	38.4	[7]
Korea	Infant (n = 71; 6–14 months)	2019	<LOD [a]	8.57	32.4	1.7	12.2	7.56	[8]
Germany	Children (n = 2256; 3–17 years)	2015–2017	23.1	26.2	21	1.5	11.1	7.7	[9]
USA	Children (n = 180; 3–6 years)	2014–2016	39	19	20	1.9	20	13	[10]
USA	Children (n = 415; 6–11 years)	2015–2016	22.1	11.6	15.4	1.30	9.00	6.10	[11]
Brazil	Children (n = 300; 6–14 years)	2012–2013	57.3	43.8	42.4	19.2	23.8	16.7	[12]
Spain [c]	Children (n = 119; 5–11 years)	2011–2012	199	61.4	NA	6.85	38.4	24.3	[13]
Japan	Children (n = 29; 3–6 years)	2009–2010	NA [b]	105	<LOD	14.1	NA	58.4	[14]
Thailand	Children (n = 104; 6–10 years)		9.1	5.0	43.6	3.8	27.3	19.1	
Indonesia	Children (n = 89; 5–11 years)	2017−2018	37.8	15.1	44.3	10.0	63.4	55.4	[15]
Saudi Arabia	Children (n = 109; 3–9 years)		268	92.4	151	7.9	51	45.7	

[a] LOD: limit of detection; [b] NA: not available; [c] geometric mean concentrations.

Figure S1 Scatter plots of Spearman's correlation between DnBP in diapers and urinary mnBP of infants (*n* = 66)

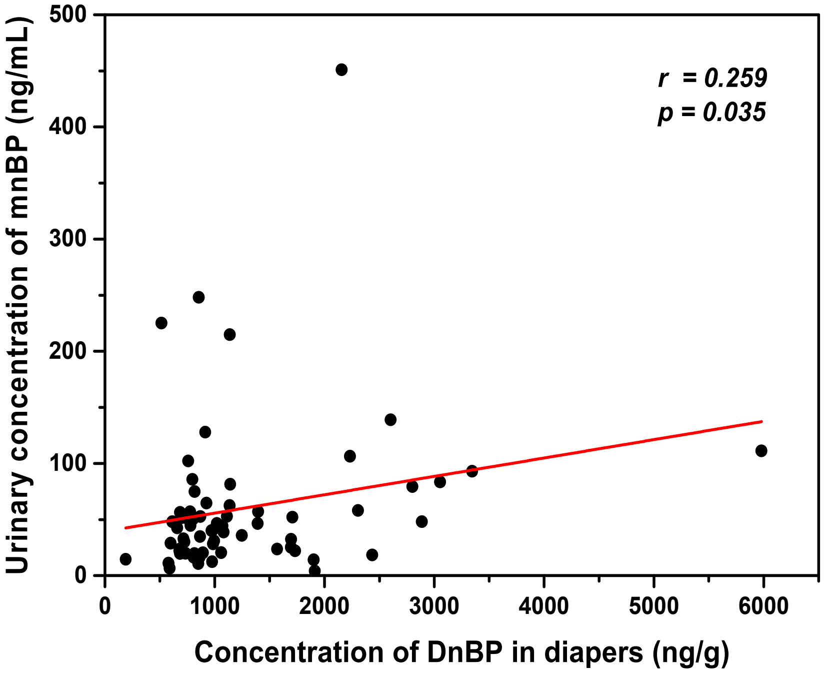

References

1. Ma, S.; Hu, X.; Tang, J.; Cui, J.; Lin, M.; Wang, F.; Yang, Y.; Yu, Y. Urinary metabolites and handwipe phthalate levels among adults and children in southern China: Implication for dermal exposure. *J. Hazard. Mat.*, **2022**, 439, 129639.
2. Kuang, H.; Li, Y.; Jiang, W.; Wu, P.; Tan, J.; Zhang, H.; Pang, Q.; Ma, S.; An, T.; Fan, R. Simultaneous determination of urinary 31 metabolites of VOCs, 8-hydroxy-2′-deoxyguanosine, and trans-3′-hydroxycotinine by UPLC-MS/MS: 13C- and 15N-labeled isotoped internal standards are more effective on reduction of matrix effect. *Anal. Bioanal. Chem.*, **2019**, 411, 7841-7855.
3. Tang, Z.; Chai, M.; Cheng, J.; Wang, Y.; Huang, Q. Occurrence and Distribution of Phthalates in Sanitary Napkins from Six Countries: Implications for Women's Health. *Environ. Sci. Tech.*, **2019**, 53, 13919-13928.
4. Ishii, S.; Katagiri, R.; Minobe, Y.; Kuribara, I.; Wada, T.; Wada, M.; Imai, S. Investigation of the amount of transdermal exposure of newborn babies to phthalates in paper diapers and certification of the safety of paper diapers. *Regul. Toxicol. Pharmacol.*, **2015**, 73, 85-92.
5. Völkel, W.; Kiranoglu, M.; Schuster, R.; Fromme, H. Phthalate intake by infants calculated from biomonitoring data. *Toxicol. Lett.*, **2014**, 225, 222-229.
6. Gao, C.-J.; Liu, L.-Y.; Ma, W.-L.; Ren, N.-Q.; Guo, Y.; Zhu, N.-Z.; Jiang, L.; Li, Y.-F.; Kannan, K. Phthalate metabolites in urine of Chinese young adults: Concentration, profile, exposure and cumulative risk assessment. *Sci. Total. Environ.*, **2016**, 543, 19-27.
7. Yu, Y.; Peng, M.; Liu, Y.; Ma, J.; Wang, N.; Ma, S.; Feng, N.; Lu, S. Co-exposure to polycyclic aromatic hydrocarbons and phthalates and their associations with oxidative stress damage in school children from South China. *J. Hazard. Mat.*, **2021**, 401, 123390.
8. Kim, J.H.; Kang, D.R.; Kwak, J.M.; Lee, J.K. Concentration and Variability of Urinary Phthalate Metabolites, Bisphenol A, Triclosan, and Parabens in Korean Mother–Infant Pairs. *Sustainability*, **2020**, 12, 8516.
9. Schwedler, G.; Rucic, E.; Lange, R.; Conrad, A.; Koch, H.M.; Pälmke, C.; Brüning, T.; Schulz, C.; Schmied-Tobies, M.I.H.; Daniels, A., et al. Phthalate metabolites in urine of children and adolescents in Germany. Human biomonitoring results of the German Environmental Survey GerES V, 2014–2017. *Int. J. Hyg. Environ. Health*, **2020**, 225, 113444.
10. Hammel, S.C.; Levasseur, J.L.; Hoffman, K.; Phillips, A.L.; Lorenzo, A.M.; Calafat, A.M.; Webster, T.F.; Stapleton, H.M. Children's exposure to phthalates and non-phthalate plasticizers in the home: The TESIE study. *Environ. Int.*, **2019**, 132, 105061.
11. CDC, 2021. Fourth National Report on Human Exposure to Environmental Chemicals: Updated Tables, March 2021. Centers for Disease Control and Prevention, U.S. Department of Health and Human Services, Atlanta, Georgia.
12. Rocha, B.A.; Asimakopoulos, A.G.; Barbosa, F.; Kannan, K. Urinary concentrations of 25 phthalate metabolites in Brazilian children and their association with oxidative DNA damage. *Sci. Total. Environ.*, **2017**, 586, 152-162.
13. Cutanda, F.; Koch, H.M.; Esteban, M.; Sánchez, J.; Angerer, J.; Castaño, A. Urinary levels of eight phthalate metabolites and bisphenol A in mother–child pairs from two Spanish locations. *Int. J. Hyg. Environ. Health*, **2015**, 218, 47-57.

14. Ait Bamai, Y.; Araki, A.; Kawai, T.; Tsuboi, T.; Yoshioka, E.; Kanazawa, A.; Cong, S.; Kishi, R. Comparisons of urinary phthalate metabolites and daily phthalate intakes among Japanese families. *Int. J. Hyg. Environ. Health*, **2015**, 218, 461-470.
15. Lee, I.; Pälmke, C.; Ringbeck, B.; Ihn, Y.; Gotthardt, A.; Lee, G.; Alakeel, R.; Alrashed, M.; Tosepu, R.; Jayadipraja, E.A., et al. Urinary Concentrations of Major Phthalate and Alternative Plasticizer Metabolites in Children of Thailand, Indonesia, and Saudi Arabia, and Associated Risks. *Environ. Sci. Tech.*, **2021**, 55, 16526-16537.
